# Importance of the site of positive surgical margin in salvage external beam radiation therapy for biochemical recurrence of prostate cancer after radical prostatectomy

**DOI:** 10.1002/cam4.1408

**Published:** 2018-03-23

**Authors:** Tairo Kashihara, Satoshi Nakamura, Akihisa Wakita, Hiroyuki Okamoto, Koji Inaba, Rei Umezawa, Satoshi Shima, Keisuke Tsuchida, Kazuma Kobayashi, Kana Takahashi, Naoya Murakami, Yoshinori Ito, Hiroshi Igaki, Hiroyuki Fujimoto, Takashi Uno, Jun Itami

**Affiliations:** ^1^ Department of Radiation Oncology National Cancer Center Hospital Tokyo Japan; ^2^ Department of Urological Oncology National Cancer Center Hospital Tokyo Japan; ^3^ Department of Radiology Chiba University Hospital Chiba Japan

**Keywords:** Prostate cancer, salvage radiotherapy, irradiation field

## Abstract

The aim of this study was to examine long‐term outcomes in patients who received salvage radiotherapy (SRT) for biochemical recurrence (BRec) of prostate cancer after radical prostatectomy (RP). One hundred and twenty patients with prostate cancer who underwent SRT for BRec after RP without evidence of clinical disease were identified in our institution from 2002 to 2014. Prescription doses to prostate beds were 64.8 Gy with a fractional dose of 1.8 Gy in 96.7% of the patients. In three‐dimensional conformal radiation therapy (3D‐CRT), the seminal vesicle bed (SVB) was not included in the radiation fields. The prognostic factors for BRec‐free survival (BRFS) and incidence of acute and late toxicities were investigated. Median follow‐up duration after SRT was 64.9 months. The 5‐year rates of BRFS, overall survival (OS), cause‐specific survival (CSS), and clinical recurrence‐free survival (CRFS) were 39.2%, 98.3%, 97.0%, and 91.9%, respectively. Only two patients experienced late grade 3 toxicity of hematuria. Multivariate analysis revealed that BRFS was significantly favorable in patients with prostate‐specific antigen (PSA) values <0.5 ng/mL at the initiation of SRT and pathological Gleason score not including Gleason grade 5. In patients treated with 3D‐CRT, a positive surgical margin at the base of the prostate influenced BRFS unfavorably in comparison with positive surgical margins at other sites. SRT for patients with BRec after RP was performed very safely in our institution. However, to improve BRFS, adequate inclusion of the SVB appears mandatory, especially in cases of positive surgical margins at the base of the prostate.

## Introduction

According to global cancer statistics in 2012, prostate cancer had the second highest estimated incidence in men, after lung cancer, and the fifth highest the mortality rate in men. Estimated age‐standard incidence and mortality rates of prostate cancer in eastern Asia were 31.1 and 7.8 per 100,000 men, respectively 1.

**Figure 1:**
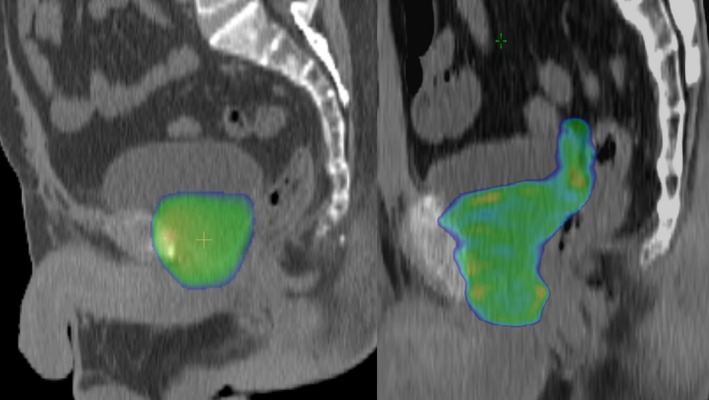
Comparison of the dose distributions of the three‐dimensional conformal radiation therapy (3D‐CRT) and intensity‐modulated radiation therapy (IMRT) in this study. Seminal vesicle bed (SVB) was not irradiated in the 3D‐CRT.

Radical prostatectomy (RP) is a curative therapy for localized prostate cancer. However, about 15–30% of patients treated with RP experience biochemical recurrence (BRec) within 5 years 2, 3. Approximately one‐third of patients with BRec after RP will have distant metastases, and the median time to the development of distant metastases following BRec is 8 years 3. Salvage radiation therapy (SRT) has been reported as an effective treatment for BRec after RP 4, 5, 6, 7, 8, 9, 10, 11, 12, 13, 14, 15, 16. Reports have varied regarding prognostic factors for biochemical control after SRT. For example, pre‐SRT prostate‐specific antigen (PSA) level, SRT dose, and pathological findings of RP specimens such as Gleason score (GS), surgical margin status (SM), seminal vesicle involvement (pSV), perineural invasion (pn), extraprostatic extension ≥2 mm, PSA doubling time (PSADT), concurrent hormone therapy, and early institution of SRT 4, 5, 6, 7, 8, 9, 10, 11, 12, 13, 14, 15, 16 have all been reported in various combinations. In our institution, intensity‐modulated radiation therapy (IMRT) has been employed in SRT for BRec after RP since 2009. Treatment planning for the IMRT has been performed in accordance with Radiation Therapy Oncology Group (RTOG) guidelines 17. Prior to the implementation of IMRT, three‐dimensional conformal radiation therapy (3D‐CRT) had been performed in SRT. However, the radiation fields of the 3D‐CRT did not include the seminal vesicle bed (SVB), as recommended in the RTOG guidelines (Fig. 1). Hence, the relationship between radiation therapy method and biochemical control was investigated in this study. Hitherto, the significance of radiation fields in SRT has rarely been reported.

## Materials and Methods

Data regarding patients with prostate cancer who had undergone RP, experienced BRec without evidence of clinical disease, and received SRT in National Cancer Center Hospital from October 2002 to June 2014 were retrieved from the radiation oncology database. All patients were examined by whole‐body computed tomography (CT) and bone scintigraphy, and no macroscopic lesions were detected prior to initiation of the salvage therapy. One hundred and twenty patients who met these criteria were retrospectively analyzed. Patients were excluded from the study if they underwent follow‐up for less than one year.

Salvage therapy after RP was instituted for BRec with a PSA value ≥0.2 ng/mL 18, 19, or in cases of patients in whom the attending urologists performed salvage therapy because PSA kept increasing from the nadir PSA value after RP despite values less than 0.2 ng/mL. In 36 patients, salvage therapy was begun with hormonal therapy (HT) and SRT followed. Other patients were treated initially with SRT with or without concurrent HT.

The associations of clinical, pathological, and therapeutic parameters with various survival types were evaluated. For patients referred from other hospitals, all operative specimens from the referring hospitals were re‐examined and reviewed pathologically in our institution.

Demographic and treatment characteristics of the 120 patients are shown in Table 1. The median follow‐up period after SRT was 62.9 months (range: 13–150 months). Median patient age at the time of RP was 61 years, and median initial PSA was 14.00 ng/mL. Regarding GS of operative specimens, 60% of the patients had GS 7, and GS of one patient was unknown. In 74 patients, GS included Gleason grade (GG) of 5. In almost half of the patients (55 patients, 45.8%), SMs were positive for cancer cells. The SM was positive at the base of the prostate in 15 patients, among whom 10 patients were treated with 3D‐CRT. Tumors were classified as T2, T3a, T3b, and T4 in 41, 41, 25, and 13 patients, respectively. T4 was diagnosed by bladder invasion in all patients.

**Table 1:** Demographic and treatment characteristics of the 120 patients

Characteristics	*n* (%)
Total number of patients	120 (100)
Median age at the RP [range]	61 years [49–76]
Median initial PSA [range]	14.00 ng/mL [3.60–73.51]
RP operative pathology	
Operative Gleason score	
≤ 6	5 (4.2)
3 + 4	21 (17.5)
4 + 3	51 (42.5)
≥8	42 (35.0)
Gleason grade with 5	74 (61.7)
Positive surgical margin	55 (45.8)
Perineural invasion	95 (79.2)
Lymphatic invasion	14 (11.7)
Extracapsular invasion	55 (45.8)
Positive lymph nodes	10 (8.3)
Seminal vesicle involvement	30 (25.0)
Pathological tumor stage	
T2	41 (34.2)
T3a	41 (34.2)
T3b	25 (20.8)
T4	13 (10.8)
Median PSA nadir after RP [range]	0.024 ng/mL [0.001–3.112]
Median interval from RP to salvage therapy [range]	739 days [44–3232]
Median age at the SRT^+^ [range]	66 years [51–77]
Median PSA at the initiation of salvage therapy [range]	0.423 ng/mL [0.091–8.172]
Median PSA doubling time before salvage therapy [range]	170.7 days [27.3–824.5]
Salvage therapy with HT	39 (32.5)
HT only before SRT	18 (15.0)
HT before and during SRT	19 (15.8)
HT only during SRT	2 (1.7)
HT after SRT	0
Median duration of HT [range]	5 months [1–58]
Radiation method	
Three‐dimensional conformal radiotherapy	87 (72.5)
Intensity‐modulated radiation therapy	33 (27.5)
Whole pelvic radiation therapy	
Done	10 (8.3)
Not done	110 (91.7)
Radiation dose	
60 Gy in 30 fractions	4 (3.3)
64.8 Gy in 36 fractions	116 (96.7)
Median PSA nadir after SRT [range]	0.012 [0.001–0.912]

HT, hormonal therapy; PSA, prostatic‐specific antigen; RP, radical prostatectomy; SRT, salvage radiation therapy.

Median interval from RP to the beginning of salvage therapy was 739 days (range: 44–3232 days). PSA nadir after RP and PSA at the initiation of salvage therapy was 0.024 ng/mL and 0.423 ng/mL, respectively. Median PSADT before salvage therapy was 170.7 days (range: 27.3–824.5 days). In 39 patients, salvage therapy after RP included HT with luteinizing hormone‐releasing hormone (LHRH) and/or antiandrogen in addition to SRT. No patients underwent HT after SRT. Median duration of the HT was 5 months (range: 1–58 months). Eighty‐one of the 120 patients did not receive HT and were treated with SRT only.

IMRT was performed in 33 patients (27.5%), whereas 3D‐CRT with seven fields was employed in 87 patients (72.5%). Both IMRT and 3D‐CRT were delivered with 15 MV X‐rays from linear accelerators (Varian, Palo Alto, CA). Prescription dose was the dose at the isocenter in 3D‐CRT, while mean dose of the planning target volume corresponded to the prescription dose in IMRT. The prescribed doses were 64.8 Gy with a fractional dose of 1.8 Gy in 116 patients (96.7%) and 60 Gy in 2 Gy fractions in four patients (3.3%). In 10 patients with pathologically positive lymph node involvement, bilateral pelvic lymph node stations up to the upper margin of L5 were also irradiated. In six patients of the 10, a dose of 45 Gy in 25 fractions to the pelvis was followed by 19.8 Gy in 11 fractions to the prostatic bed. In the remaining four patients, the simultaneous integrated boost IMRT (SIB‐IMRT) technique was used, consisting of prostatic bed irradiation of 64.8 Gy and bilateral pelvic lymph node irradiation of 52.2 Gy, both in 36 fractions.

RTOG guidelines proposed that the superior edge of the clinical target volume (CTV) should be level with the cut end of the vas deferens or 3–4 cm above the top of the symphysis, or the CTV should include seminal vesicle remnants in cases of pathological evidence of seminal vesicle involvement. 17 In the current patient series, the CTV of the IMRT was contoured in accordance with the RTOG guidelines; however, the SVB was included in the CTV in all patients, regardless of pathological tumor invasion of the seminal vesicles. On the other hand, the CTV of the 3D‐CRT included only the prostatic bed, and the SVB was not included in the radiation fields (Fig. 1) 20.

BRec after SRT was defined as two consecutive PSA values **≥**0.2 ng/mL with the second date considered as the time of BRec after SRT. Complications due to SRT were evaluated according to the Common Terminology Criteria for Adverse Events (CTCAE) ver.4.0. Late toxicity was defined as morbidities occurring more than three months after SRT.

BRec‐free survival (BRFS), overall survival (OS), cancer‐specific survival (CSS), and clinical recurrence‐free survival (CRFS) were calculated according to the Kaplan–Meier method with the last date of SRT assumed as day 0. The log‐rank test was applied to identify statistical differences. In the calculation of BRFS, BRec (two consecutive PSA values **≥**0.2 ng/mL) and the initiation of HT were considered as an event, with death without BRec treated as censored. In calculating CSS and CRFS, deaths from causes other than prostate cancer were treated as censored. Multivariate Cox proportional hazards regression models were used to identify independent factors influencing BRFS after SRT. Variables with *P*‐values <0.05 in the univariate analysis were selected for the multivariate analysis. *P*‐values <0.05 were considered statistically significant. Statistical analysis was performed with IBM SPSS Statistics (v19.0.0; IBM Corp., Armonk, NY).

## Results

Acute adverse events of grade **≥**3 were not observed. Late genitourinary (GU) events of grade 3 hematuria were observed in two patients at 67 and 110 months, respectively, after SRT. Neither patient took any anticoagulant or antiplatelet agents. Both patients received transurethral electrocoagulation, and one patient underwent hyperbaric oxygen therapy as well. No late gastrointestinal (GI) events of grade >3 were observed.

**Figure 2:**
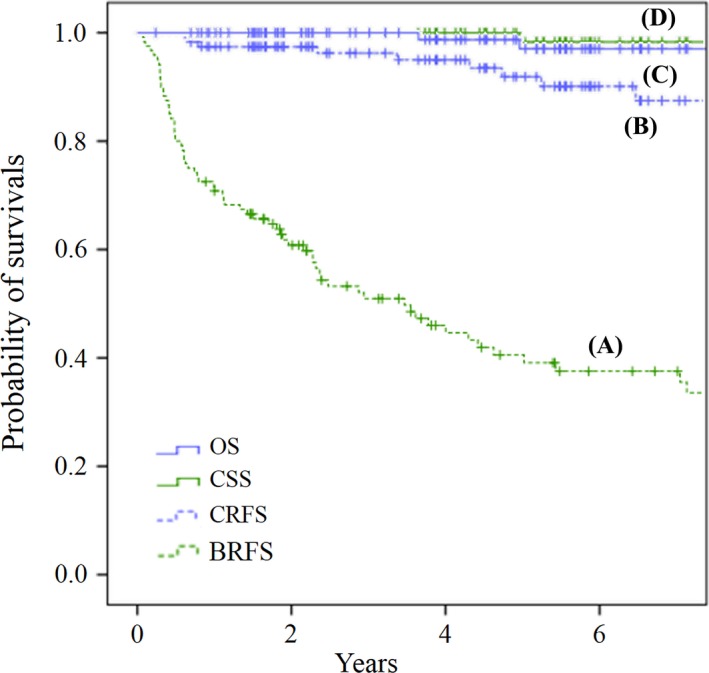
Biochemical recurrence‐free survival (BRFS), overall survival (OS), cancer‐specific survival (CSS), and clinical recurrence‐free survival (CRFS) of all patients. The 5‐year rates of BRFS, OS, CSS, and CRFS were 39.2%, 98.7%, 100%, and 91.9%, respectively.

Five‐year probabilities of BRFS, OS, CSS, and CRFS were 39.2%, 98.3%, 97.0%, and 91.9%, respectively (Fig. 2). After SRT, BRec was observed in 65 patients, and HT was initiated in two patients before the diagnosis of BRec was established. Five patients died during the follow‐up period, among whom three died of prostate cancer and the remaining two died of other cancers (renal and pancreas cancers). Clinical recurrences were detected in nine patients. Seven patients experienced bone metastasis, and one also had liver metastasis. Pelvic lymph node and subcutaneous metastases were each observed in one patient.

**Figure 3:**
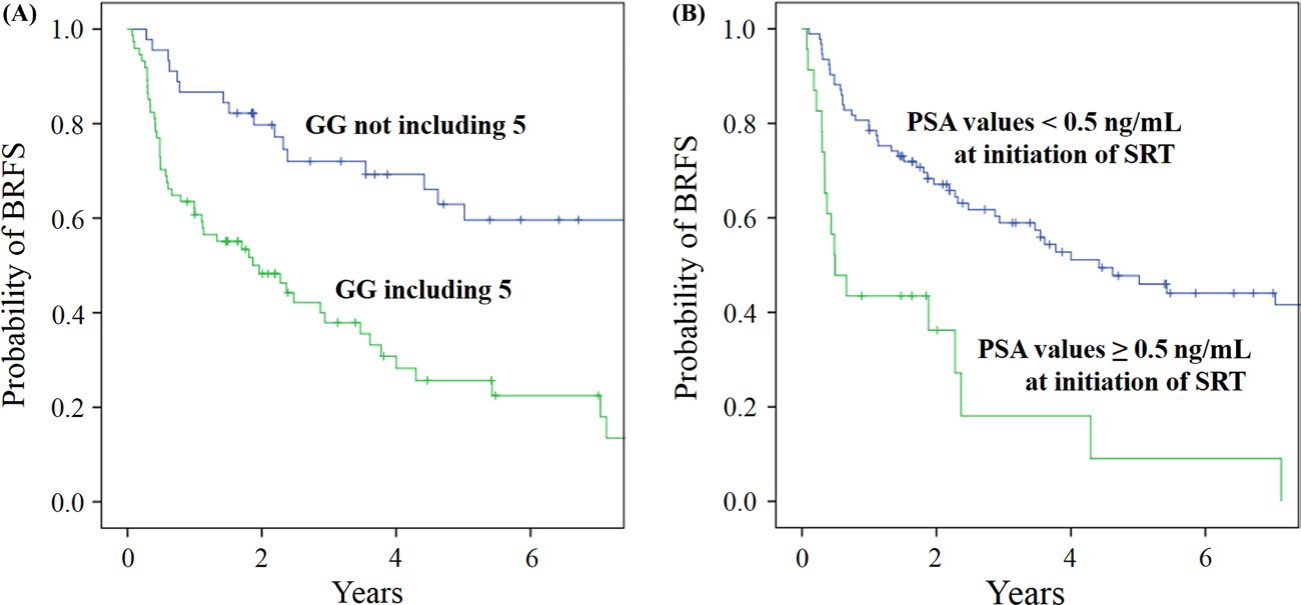
Biochemical recurrence‐free survivals (BRFS) according to the PSA values at the initiation of salvage radiation therapy (SRT) and the presence of Gleason grade (GG) 5 in Gleason score (GS).

In univariate analysis, BRFS was favorable with statistically significant differences in patients with PSA <0.5 ng/mL at the initiation of salvage therapy (*P *=* *0.005, 5‐year BRFS 48.8% with PSA <0.5 ng/mL vs. 25.9% with PSA 0.5 ng/mL); PSA values <0.5 ng/mL at the initiation of SRT (*P *<* *0.001, 5‐year BRFS 47.8% with PSA <0.5 ng/mL vs. 9.1% with PSA **≥**0.5 ng/mL); positive SMs (*P *=* *0.001, 5‐year BRFS 64.3% with positive SM vs. 23.5% with negative SM); GG not including 5 (*P *<* *0.001, 5‐year BRFS 62.9% with GS not including GG 5 vs. 25.7% with GS including GG 5); GS **≥**7 (*P *=* *0.002, 5‐year BRFS % 49.0% with GS <7 vs. 25.3% with GS **≥≥**8); and IMRT (*P *=* *0.045, 5‐year BRFS 56.6% with IMRT vs. 36.5% with 3D‐CRT) (Fig. 3).

Pathological T‐stage **≥**3 (*P *=* *0.067, 5‐year BRFS 47.4% with T < 3 vs. 37.3% with T **≥** 3) was an unfavorable factor with marginal significance.

On the other hand, SV involvement, positive lymph nodes, extracapsular invasion, lymphatic invasion, perineural invasion, PSA nadir after RP, administration of HT in salvage therapy, and PSADT had no statistically significant influence on BRFS (Table 2).

**Table 2:** Univariate and multivariate analyses of possible clinical factors predicting biochemical recurrence‐free survival (BRFS)

Factor	Univariate				Multivariate		
	*P*‐value	HR	95% CI	Favorable factor	*P*‐value	HR	95% CI
PSA at the initiation of salvage therapy	0.005	0.498	0.303–0.818	PSA <0.5 ng/mL			
PSA at the initiation of SRT	<0.001	0.305	0.174–0.533	PSA <0.5 ng/mL	0.005	0.432	0.241–0.773
Surgical margin	0.001	2.411	1.426–4.075	Positive	0.218	1.444	0.805–2.590
Presence of Gleason grade 5	<0.001	0.304	0.171–0.540	No Gleason grade 5	0.001	0.330	0.174–0.626
Gleason Score	0.002	0.467	0.284–0.767	Gleason score ≤7			
IMRT or 3D‐CRT	0.045	0.490	0.240–0.999	IMRT	0.065	2.014	0.959–4.233
Pathological T‐stage	0.067	0.615	0.364–1.039	T < 3			
PSA nadir after SRT	<0.001	7.388	4.391–12.430	PSA <0.05 ng/mL			
Seminal vesicle involvement	0.684						
Positive lymph node	0.145						
Extracapsular invasion	0.171						
Lymphatic invasion	0.145						
Perineural invasion	0.280						
PSA nadir after RP	0.811						
Neoadjuvant HT	0.450						
Concurrent HT	0.486						
PSA doubling time	0.189						

CI, confidence interval; HR, hazard risk; HT, hormonal therapy; IMRT, intensity‐modulated radiation therapy; PSA, prostate‐specific antigen; RP, radical prostatectomy; SM, surgical margin; SRT, salvage radiation therapy; 3D‐CRT, 3‐dimensional conformal radiation therapy.

As a prognostic factor after the completion of SRT, PSA nadir after SRT was revealed to influence BRFS with statistical significance, with PSA nadir <0.05 showing favorable outcome (*P *<* *0.001, 5‐year BRFS 61.6% with PSA nadir after SRT <0.05 vs. 7.6% with PSA nadir after SRT **≥**0.05).

Multivariate Cox proportional hazards analysis included all possible variables prior to SRT (excluding PSA nadir after SRT) with *P *<* *0.05 in the univariate analysis. However, only a factor with a lower *P*‐value was selected in the strongly interrelated prognostic factors. PSA values at the initiation of SRT and salvage therapy are strongly correlated; therefore, PSA at the initiation of SRT was selected. Moreover, because GS and the presence of GG 5 are interrelated with a lower *P*‐value in the presence of GG 5, the presence of GG 5 was included in the multivariate analysis. The multivariate analysis revealed that the presence of GG 5 and PSA values **≥**0.5 ng/mL at the initiation of SRT was unfavorable prognostic factors for BRFS with statistical significance and for 3D‐CRT with marginally statistical significance (Table 2).

**Figure 4:**
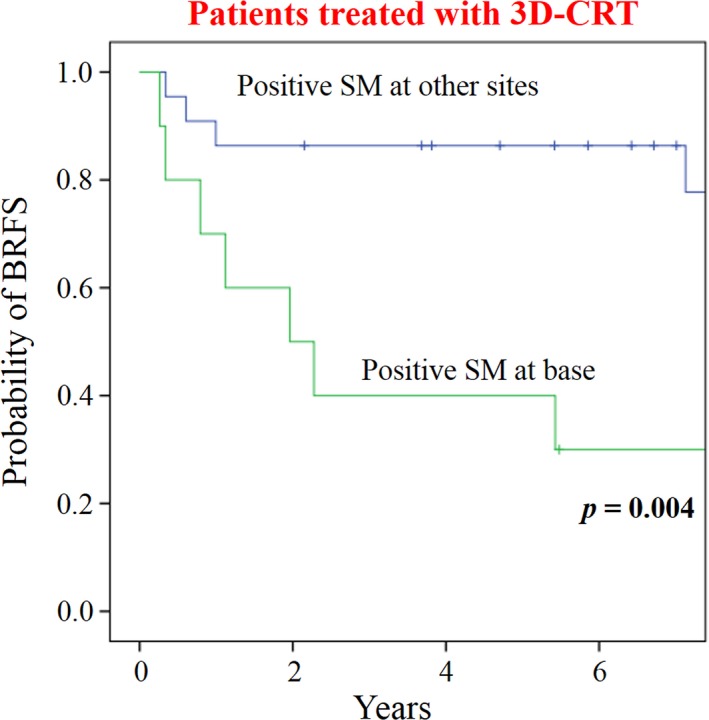
Biochemical recurrence‐free survivals (BRFS) by the sites of positive surgical margin (SM) in patients treated with three‐dimensional conformal radiation therapy (3D‐CRT).

Among patients treated with 3D‐CRT, 32 had positive SMs. Ten patients had positive SMs at the base of the prostate, and 22 had positive SMs at other sites. BRFS was significantly different between these two patient groups (*P *=* *0.004) (Fig. 4). Five‐year BRFS was 40.0% and 86.4% in the patients with positive SMs at the base and at the other sites, respectively. In the 33 patients treated with IMRT, no difference was observed in BRFS according to the site of positive SM, partially because of the low number of patients undergoing IMRT.

## Discussion

In this study, among patients without evidence of clinical disease at the initiation of salvage therapy, GS not including GG 5 and PSA values <0.5 ng/mL at the initiation of SRT was proven to be favorable prognostic factors for BRFS by SRT after RP with statistical significance, and usage of IMRT favorably influenced BRFS with marginal significance. These results were largely the same as those described in previous reports. However, in the present report, the relationship between BRFS and radiation dose, which has been demonstrated in past reports, was not analyzed because almost all patients received the same dose of 64.8 Gy. Radiation dose has been reported as an important prognostic factor 10, 11, 12, 13, 14, 15, 16, 17. According to Bernard et al. 11, in a high‐dose group (radiation dose >66.6 Gy), BRFS was higher than in the low‐ (radiation dose <64.8 Gy) and moderate (radiation dose; 64.8–66.6 Gy)‐dose groups. In a recent study 16, SRT doses **≥**68 Gy were associated with a reduced risk of BRec. The 5‐year rates of BRFS with SRT doses of less than 66 Gy, 66–67.99 Gy, 68–71.99 Gy, and **≥**72 Gy were 46%, 44%, 53%, and 61%, respectively. Moreover, a systematic review and meta‐analysis by King et al. 21 demonstrated a dose–response relationship between SRT dose and BRFS. A well‐fit sigmoidal relationship of dose and biochemical control showed 50% PSA control at a dose of 65.8 Gy. The results of this high‐dose irradiation were better than the BRFS achieved in this study, and dose increment seems mandatory to improve BRFS of SRT for BRec after PR.

To our knowledge, this is the first report that investigates the prognostic significance of the site of positive SMs. When all patients with a positive SM were analyzed, it was of marginal significance for BRFS whether the positive SM was at the base or at other sites. However, in patients treated with 3D‐CRT, those with a positive SM at the base of the prostate showed poorer BRFS with statistical significance. In the current series, SRT by 3D‐CRT did not include the SVB, in contrast to IMRT, which included the SVB (Fig. 1). It seems probable that this inadequacy of irradiation fields at the SVB in the 3D‐CRT caused poorer BRFS, especially in the patients with a positive SM at the base, which lies in the cranial part of the prostate bed. These findings indicate that SVB should also be included in the CTV in patients with a positive SM at the base, even in cases in which pathological SV invasion is not obvious. The findings also emphasize the importance of the cranial margins in SRT for BRec after RP.

In RTOG 9601 22, in which SRT of 64.8 Gy, a dose similar to that of the current series, was delivered to the BRec after PR, 5‐year BRFS without antiandrogen therapy reached almost 50%, although only patients with less advanced disease, with pT2 and pT3, were recruited in RTOG 9601 in contrast to the current series, and a different definition of BRec was used. Poor BRFS in the current series could be partially caused by the inadequate radiation fields in 3D‐CRT.

Even in comparison with the results of the Japanese series of SRT for BRec after RP reported by Mizowaki et al., the 5‐year BRFS in the present series was unfavorable (39.2% in the current series vs. 50.1% in the study by Mizowaki, et al.) 9. In Mizowaki's series, 40.9% of the patients received radiation doses of more than 65 Gy. Poorer results in the current series seem to be caused by the lower SRT dose and the inadequate radiation fields.

Compared to previous reports, a lower incidence of late adverse events was observed in the present study. The reasons for this lower incidence of late adverse events are unknown, but the routine employment of 15 MV X‐ray beams could be contributing in the reduction in rectal and bladder doses 23. Implementation of IMRT further reduced doses to the organs at risk and the incidence of adverse events 24, 25.

There are several limitations in this study. Firstly, the follow‐up period of patients receiving IMRT was shorter than that of patients receiving 3D‐CRT. Further follow‐up of the patients undergoing IMRT is mandatory to confirm the superiority of IMRT in BRFS. Secondly, there exists some inhomogeneity concerning HT and PSA values before delivery of the salvage therapy. In some patients, salvage therapy was performed before their PSA values were higher than 0.2. Additionally, the length of HT ranged from 1 month to 58 months. However, multivariate analysis took all of these factors into account, which could have partially compensated for the inhomogeneity. Lastly, this report is a retrospective study and could include some unknown biases.

In conclusion, this retrospective study found that SRT can be performed safely and that the presence of GG 5 and PSA values **≥**0.5 ng/mL at the initiation of SRT was unfavorable prognostic factors for BRFS. The inadequacy of radiation fields in the SVB affected BRFS adversely, especially in patients with a positive SM at the base of the prostate. Irradiation sufficiently including the SVB and dose escalation seems to be very important to improve the BRFS of SRT.

## Conflict of Interest

The authors declare that authors have no conflict of interests.
